# Clinical Presentation, Risk Factors, and Clinical Outcomes of Patients Who Underwent Major Upper-Limb Amputation for Nontraumatic Indications: A Retrospective Study

**DOI:** 10.7759/cureus.105844

**Published:** 2026-03-25

**Authors:** Ravi S, Spoorthy Srinivas, Manjunath B D

**Affiliations:** 1 Department of General Surgery, Bangalore Medical College and Research Institute, Bengaluru, IND

**Keywords:** acute limb ischemia, necrotizing fasciitis, nontraumatic upper limb amputation, peripheral arterial disease, surgical outcomes, upper limb infections, vascular insufficiency

## Abstract

Background

Major upper-limb amputations are performed for various nontraumatic causes such as limb ischemia, malignancy, and infections. These procedures have significant psychosocial and functional impacts on patients, often leading to long-term disability and reduced quality of life. Understanding the interplay between the risk factors and disease progression is essential for improving preventive and therapeutic strategies. Limited data exist correlating clinical profile and surgical outcomes in nontraumatic major upper-limb amputations. This study aims to evaluate these factors in patients undergoing nontraumatic major upper-limb amputation at a tertiary care center. This study descriptively analyzes the demographic details, clinical presentation, risk factors, and surgical outcomes of patients undergoing major upper-limb amputation for nontraumatic indications.

Methodology

A retrospective observational study was conducted over a two-year period (August 2023 to August 2025) in the Department of General Surgery at a tertiary care center. In total, 23 patients who underwent major upper-limb amputation for nontraumatic causes were included. Data were collected from hospital records with respect to demographic details, comorbidities, Doppler and CT angiography findings, indications and level of amputation, and postoperative outcomes, including duration of hospital stay, revision amputation, surgical site infection, intensive care unit admission, readmission, and mortality.

Results

Among the 23 patients, 78.9% were middle-aged, and 69.6% were male. The left upper limb was involved in 73.9% of cases. Diabetes mellitus was present in 60.9% of patients, hypertension in 39.1%, and 65.2% were smokers. Anemia was observed in 69.5%, and hypoalbuminemia in 39%. Brachial artery involvement was noted in 30% of cases. Limb ischemia was the most common indication (78%), followed by necrotizing fasciitis (17.4%). Above-elbow amputation was performed in 91.3% of patients. Revision amputation was required in 8.6% of cases. Hospital readmission occurred in 4.3%, and one (4.3%) case of mortality was recorded. The mean hospital stay was 8.7 days.

Conclusions

Vascular insufficiency was noted to be the leading cause of nontraumatic major upper-limb amputation. While limited by a small sample size, these findings provide a descriptive profile with respect to nontraumatic major upper-limb amputations. Early diagnosis and aggressive management of vascular and infectious etiologies may improve limb salvage rates and reduce morbidity and mortality.

## Introduction

Major upper-limb amputation refers to the surgical removal of the upper extremity proximal to the wrist joint, including transradial, transhumeral, and forequarter amputations and shoulder disarticulation [1]. While most upper-limb amputations worldwide are performed for traumatic injuries, a smaller yet clinically significant proportion occur for nontraumatic causes, such as infection, acute limb ischemia, and malignant disease [2,3]. These procedures have devastating effects on the patient’s physical function, social integration, and psychological well-being.

Globally, upper-limb amputations account for only 8%-10% of all amputations, far lower than lower-limb amputations; however, their disability burden per patient is substantially higher [4]. The worldwide prevalence of limb loss due to traumatic causes alone was estimated at 552 million individuals as of 2019, with 13.2 million new cases annually [5]. In South Asia, the burden of years lived with disability due to amputation is the highest in the world, with India contributing the largest share [6]. Indian hospital-based studies have indicated that upper-limb amputations constitute roughly 5%-9% of all amputations, with nontraumatic causes forming a minority but increasing steadily [7,8].

The pattern of etiology in nontraumatic upper-limb loss has evolved alongside demographic and epidemiologic transitions. Aging populations and the rising prevalence of diabetes mellitus, hypertension, and peripheral arterial disease have increased the risk of critical limb ischemia and uncontrolled soft-tissue infections [9]. In 2010, diabetes and peripheral arterial disease together accounted for over half of all nontraumatic upper limb and lower limb amputations worldwide [3]. Smoking, chronic kidney disease, and malnutrition further exacerbate tissue hypoxia and impair wound healing, leading to limb loss [10].

Upper-limb infections remain a major cause of amputation in developing regions. Lim et al. [11] studied 244 patients with upper-limb infections and found that individuals with diabetes were more likely to present emergently, harbor polymicrobial flora, and require amputation compared with those without diabetes. Necrotizing soft-tissue infections (NSTIs) of the upper extremity, though uncommon, carry a high risk of limb loss and mortality. In Uehara et al.’s [12] study of 116 patients with upper-extremity NSTI, diabetes and sepsis were independent predictors of amputation, while advanced age and renal dysfunction predicted death. Similarly, Nawijn et al. [13] reported a 14% amputation rate and 11% mortality among 122 patients with upper-limb NSTI, underscoring the grave prognosis of delayed presentation.

Ischemic etiologies for upper-limb amputations, though less frequent than for the lower-limb counterparts, remain clinically significant. Deguara et al. [14] reviewed two decades of upper-limb revascularization and found that thromboembolism (35%), trauma (31%), and chronic ischemia (17%) were the predominant causes; despite modern vascular techniques, mortality remained at 8.7%. Patients with upper-limb acute ischemia often present late due to collateral circulation masking early symptoms, leading to irreversible tissue necrosis by the time of diagnosis. In such cases, amputation is lifesaving yet profoundly disabling.

In addition to medical factors, psychosocial and functional sequelae following upper-limb loss are severe. Studies have reported higher rates of depression, anxiety, and social withdrawal among upper-limb amputees than among lower-limb amputees, primarily due to the loss of hand function and challenges in performing activities of daily living [15]. Prosthetic rehabilitation for upper limbs also lags behind that for lower limbs, particularly in low- and middle-income countries, owing to limited availability of myoelectric devices and poor affordability [16].

Using a retrospective cohort, Ting et al. [17] analyzed 140 nontraumatic upper-extremity amputations over 13 years and found that diabetes, smoking, coronary artery disease, and chronic renal failure were the major risk factors, with infection being the most common indication. However, their series reported only 5.7% major upper-limb amputations, highlighting the rarity of these cases. The distinct patient demographics, comorbidity profiles, and delayed presentation patterns in India necessitate dedicated regional analyses to guide preventive and perioperative strategies.

Despite the clinical and societal impact, most existing research focuses predominantly on lower-limb amputations and traumatic upper-limb amputations, where mechanisms, reconstructive options, and outcomes differ greatly from nontraumatic upper-limb cases [2]. Nontraumatic amputations often occur in medically compromised individuals, and perioperative management requires a nuanced understanding of systemic disease, infection control, and wound optimization. Moreover, there is a paucity of structured studies integrating clinical characteristics and surgical outcomes in this subgroup, particularly from Indian tertiary care centers.

Early recognition of upper-limb infections, timely revascularization in ischemic limbs, and regular debridement play a pivotal role in preventing progression to amputation. When amputation becomes inevitable, a comprehensive evaluation of preoperative risk factors and postoperative outcomes is essential to identifying modifiable determinants of morbidity and mortality.

Hence, this study was designed to address this knowledge gap. Through a retrospective analysis of patients undergoing major upper-limb amputation for nontraumatic causes at a tertiary care hospital, we aim to correlate clinical features, comorbid risk factors, and surgical outcomes, thereby contributing to the limited existing data on nontraumatic upper-limb amputations.

## Materials and methods

This study aims to descriptively analyze the demographic details, clinical presentation, risk factors, and surgical outcomes of patients undergoing major upper-limb amputation for nontraumatic indications. This retrospective observational study was conducted in the Department of General Surgery at Victoria Hospital, which is attached to Bangalore Medical College and Research Institute. The study was conducted over a two-year period from August 2023 to August 2025. The study included patients who underwent major upper-limb amputation for nontraumatic indications during the study period.

Ethical clearance was obtained from the Institutional Ethics Committee before data collection (approval number: BMCRI/EC/32/2025). The study was conducted in accordance with established ethical guidelines. As this was a retrospective, record-based study, strict patient confidentiality was maintained throughout, and no direct patient interaction was involved at any stage.

The study population comprised all adult patients aged 18 years and above who underwent major upper-limb amputation above the level of the wrist for nontraumatic causes during the specified study period. Patients who underwent amputation due to traumatic causes were excluded from the study. In addition, patients with incomplete or inadequate medical records were also excluded to ensure the accuracy and reliability of the collected data. A total of 23 patients satisfied the inclusion criteria and were included in the final analysis.

Data were collected retrospectively from hospital medical records, operative notes, laboratory databases, and radiological reports. Demographic and clinical parameters recorded included the patient’s age, gender, and occupation. Information regarding comorbid conditions such as diabetes mellitus, hypertension, and chronic kidney disease was documented. Smoking and alcohol history was recorded as a binary categorical variable (present/absent) based on the documentation within the clinical admission notes or social history records.

Hematological and biochemical parameters at the time of admission were recorded for all patients. These included hemoglobin levels, total leukocyte count, serum creatinine, and serum albumin levels. The presence of anemia (hemoglobin <10 g/dL) and hypoalbuminemia (serum albumin <3.5 g/dL) was determined based on standard laboratory reference values. Pus culture and sensitivity reports were analyzed in a subset of patients with necrotizing fasciitis. Antibiotic susceptibility was determined using automated VITEK-2 systems, and results were interpreted according to the Clinical and Laboratory Standards Institute (CLSI) guidelines. Multi-drug resistance was defined as non-susceptibility to at least one agent in three or more antimicrobial categories.

Radiological assessment included evaluation of arterial Doppler studies to determine the level and severity of arterial involvement. CT angiography of the affected upper limb was reviewed to assess the extent of vessel occlusion, the level of arterial compromise, and the presence or absence of collateral circulation.

Operative and postoperative details were also documented. The indication for amputation and the level at which the amputation was performed were recorded. The duration of hospital stay was noted for each patient. Postoperative outcomes were assessed by documenting complications such as surgical site infection, the need for revision amputation, admission to the intensive care unit (ICU), hospital readmission, and mortality. Postoperative outcomes were defined using standardized criteria. Surgical site infection was classified according to the Centers for Disease Control and Prevention guidelines. Revision amputation was defined as any subsequent surgical procedure to proximalize the amputation level. ICU admissions during the course of the hospital stay were noted. Hospital readmission was tracked for 30 days post-discharge, and mortality was defined as any death occurring during the primary hospital stay.

Statistical analysis

The collected data were entered into a Microsoft Excel (Microsoft Corp., Redmond, WA, USA) spreadsheet and subsequently analyzed using SPSS software, version 27 (IBM Corp., Armonk, NY, USA). Descriptive statistics were used to summarize the data. Continuous variables were expressed as means and standard deviations (SDs), while categorical variables were expressed as frequencies and percentages.

## Results

A total of 23 individuals underwent major upper-limb amputation for nontraumatic indications during the study period. Categorical variables are expressed in terms of frequency (n) and percentage (%). Continuous variables are expressed in terms of mean and SD.

Age

The age group ranged from 26 to 72 years. The group had a middle-aged to older profile, with an average (mean) age of 53.4 years. A significant proportion of the group fell within the age range of approximately 42 to 65 years. The distribution reveals that the largest segment, comprising 39.1% of the group, was concentrated within the 51-60-year age bracket. The youngest (21-30 years old) and oldest (71-80 years old) cohorts were the smallest, each representing only 8.7% of the total, as shown in Table 1.

**Table 1:** Age group distribution of the study participants.

Age group (years)	Frequency	Percent
21–30	2	8.7
31–40	1	4.3
41–50	5	21.7
51–60	9	39.1
61–70	4	17.4
71–80	2	8.7
Total	23	100.0

Gender

Males constituted a significant majority, representing 69.6% of the total, which corresponded to 16 individuals. In contrast, females were a distinct minority, comprising just 30.4% of the group, or seven individuals.

Side of the limb involved

The left upper limb was predominantly involved (73.92%) among the 23 patients who underwent major upper-limb amputation for nontraumatic indications.

Occupation

The participants had various occupational backgrounds, as shown in Table 2.

**Table 2:** Occupation of the study participants.

Occupation	Frequency	Percent
Coolie	7	30.4
Farmer	5	21.7
Homemaker	4	17.4
Construction worker	2	8.69
Flower seller	1	4.34
Driver	1	4.34
Weaver	1	4.34
Not known	2	8.69

Risk factors

The comorbidity profile of this patient group was dominated by metabolic and cardiovascular conditions, with diabetes mellitus being the most prevalent, affecting a majority of (60.9%, 14 out of 23) of individuals (Table 3). Hypertension was noted in 39.1% of patients. The high prevalence of these two conditions is highly significant, as both diabetes and hypertension are leading risk factors for the development and acceleration of peripheral vascular disease and are strongly linked to poor outcomes in vascular complications, which aligns perfectly with the primary diagnoses and high amputation rate observed in this cohort. Other isolated risk factors are shown in Table 4.

**Table 3:** Comorbidities of the study participants.

Comorbidities	Present	Absent
Diabetes mellitus	14 (60.9%)	9 (39.1%)
Hypertension	9 (39.1%)	14 (60.9%)
Ischemic heart disease	3 (13.0%)	20 (87.0%)
Cardiovascular disease	1 (4.3%)	22 (95.5%)
Hypothyroidism	1 (4.3%)	22 (95.5%)

**Table 4:** Other risk factors noticed among study participants.

Other risk factors	N (%)
Transbrachial embolectomy	2 (8.68%)
Carcinoma	2 (8.68%)
Cervical rib	1 (4.34%)
Prior limb amputations	1 (4.34%)
Recent myocardial infarction	1 (4.34%)
Deep vein thrombosis	1 (4.34%)

An analysis of the habits within the patient cohort revealed a notably high prevalence of smoking, with 15 (65.2%) individuals identified as smokers. In contrast, alcohol use was reported by a smaller, though still substantial, proportion of the group, with 10 (43.5%) individuals indicating alcohol use. Smoking is a well-established primary risk factor for the development and worsening of peripheral vascular disease, which was the most common diagnosis in this group. Due to the retrospective nature of the study, limited data were available regarding the duration of smoking among participants. No habits of intravenous drug abuse were noted among the participants.

Hematological parameters

The hemoglobin levels ranged from a concerning low of 6.65 g/dL to a normal high of 17.40 g/dL, with a group average of 11.59 g/dL, which sits at the lower end of the typical normal range. The substantial SD of 2.91 suggests significant disparity in these values within the group, indicating the presence of individuals with both anemia and normal erythrocyte levels. Similarly, the total white blood cell count exhibited an extremely wide range, from a normal 4,600 cells/mm³ to a markedly elevated 37,400 cells/mm³. In contrast, the creatinine and albumin levels showed more constrained variability, as shown in Table 5.

**Table 5:** Hematological laboratory parameters.

Laboratory parameters	N	Minimum	Maximum	Mean	SD
Hemoglobin	23	6.65 g/dL	17.40 g/dL	11.588	2.908
Total count	23	4,600 cells/mm³	37,400 cells/mm³	16,061.74	8,928.09
Creatinine	23	0.40 mg/dL	1.70 mg/dL	0.809	0.365
Albumin	23	1.5 g/dL	4.4 g/dL	3.291	0.6295

Pus culture and sensitivity

Four cases of necrotizing fasciitis were noted. Among the subset of patients, *Staphylococcus aureus* was isolated in three samples, and *Enterococcus* species was isolated from one sample. These isolates showed resistance to penicillins, third-generation cephalosporins, and fluoroquinolones. Due to the retrospective nature of the study, sensitivity testing was limited to the specific panels requested by the treating clinical team.

Level of the vessel involved

**Figure 1:**
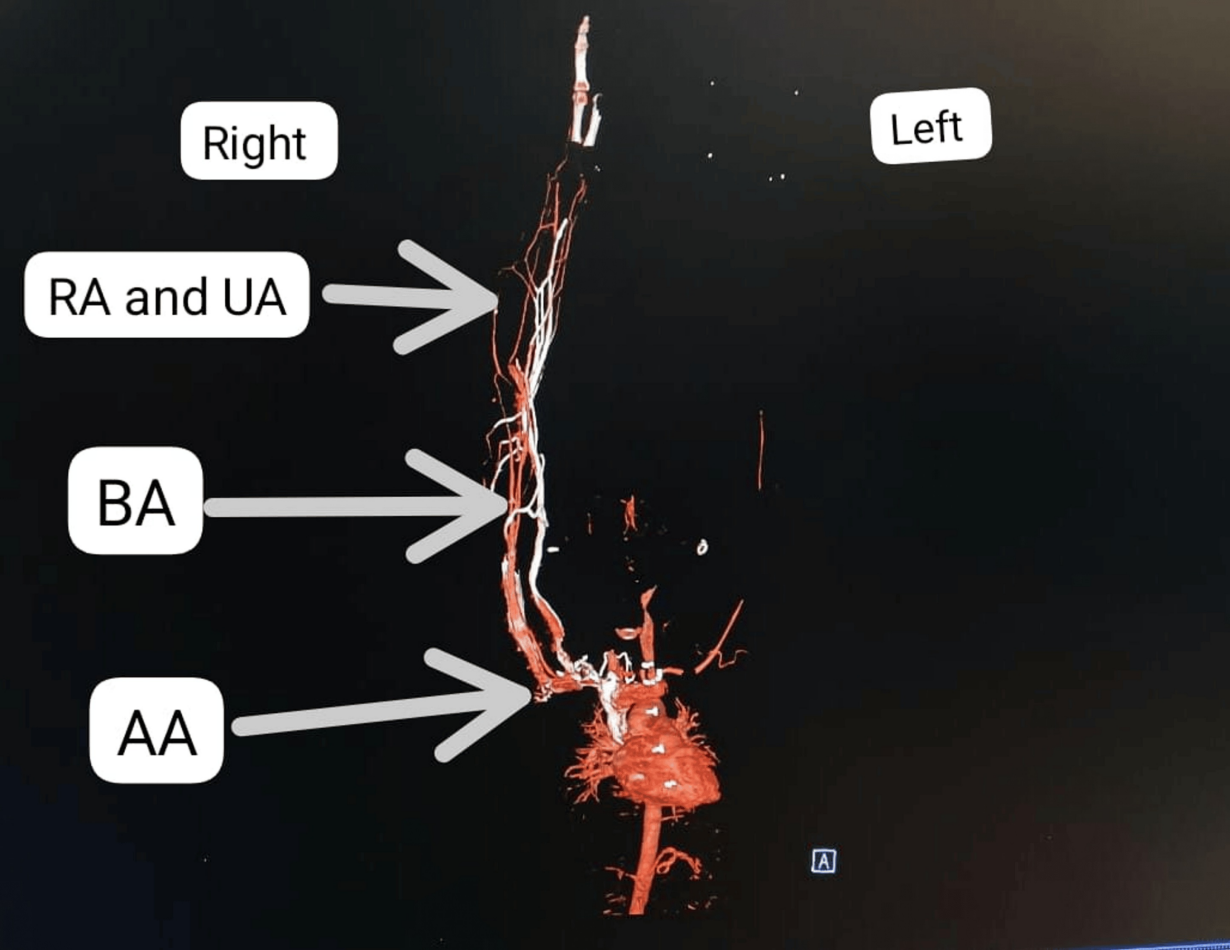
Volume-rendered CT angiographic study of the bilateral upper limbs in the coronal section, depicting near-complete thrombosis of the left subclavian and axillary arteries and complete thrombosis of the left radial and ulnar arteries. RA = radial artery; UA = ulnar artery; BA = brachial artery; AA = axillary artery

**Figure 2:**
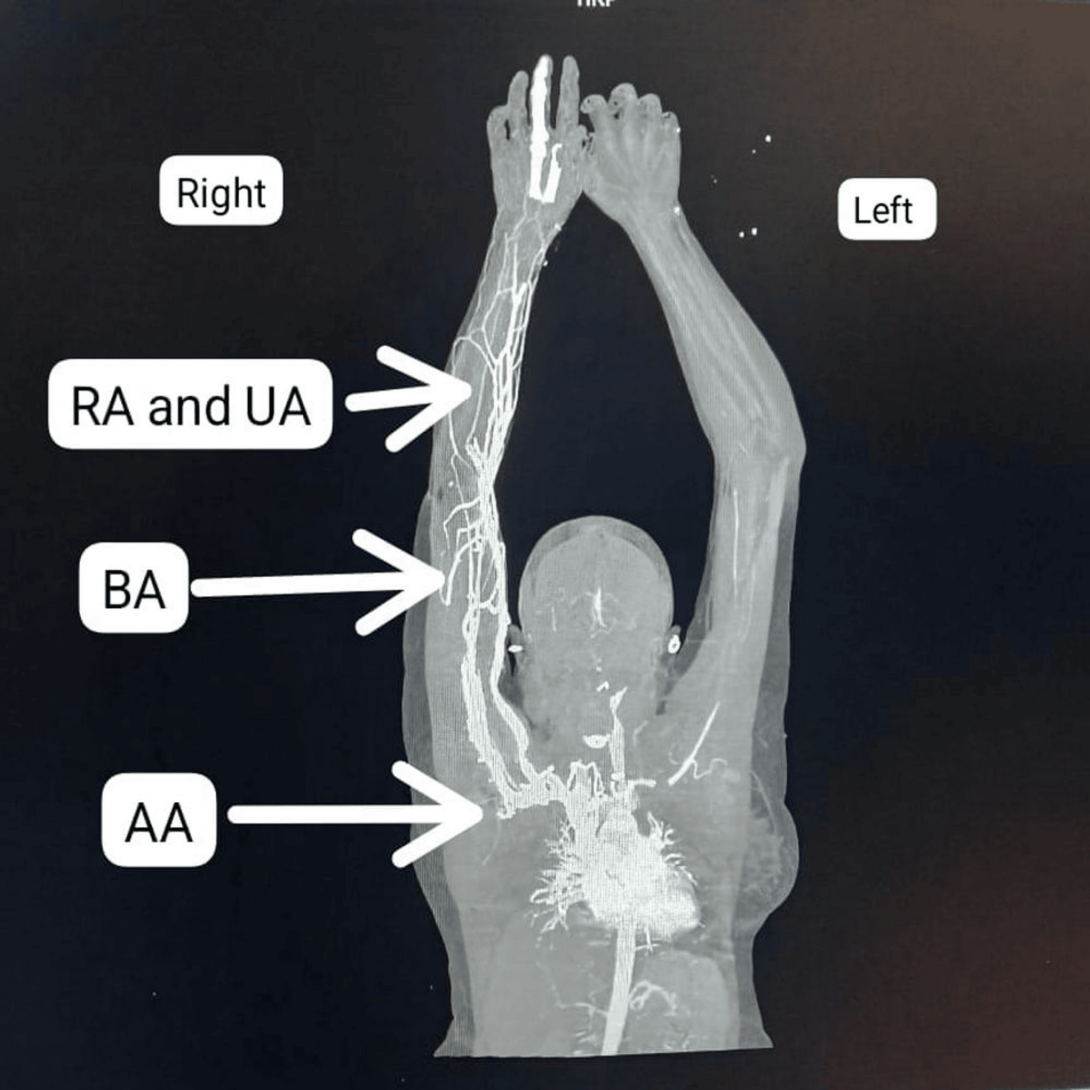
Coronal CT angiographic study of the bilateral upper limbs processed to an anatomical overlay image depicting near-complete thrombosis of the left subclavian and axillary arteries and complete thrombosis of the left radial and ulnar arteries. RA = radial artery; UA = ulnar artery; BA = brachial artery; AA = axillary artery

The brachial artery was involved in 30.43% of the patients and was the most commonly affected blood vessel, followed by the axillary (26.08%), radial and ulnar (21.73%), and subclavian (13.04%) arteries. Multivessel involvement was noted in all cases. Figure 1 and Figure 2 present the CT angiography of the bilateral upper limbs with left upper limb involvement.

Indication of upper-limb amputation

Major upper-limb amputation for vascular insufficiency accounted for 78.2% of the cases. The most prevalent diagnosis by a significant margin was upper-limb peripheral vascular disease, accounting for nearly half of all cases (52.2%), followed by upper-limb acute limb ischemia (26.0%). The remaining cases consisted of serious complications, including necrotizing fasciitis (17.4%) and compartment syndrome (4.4%), as shown in Table 6.

**Table 6:** Indications for upper-limb amputation. PVD = peripheral vascular disease

Diagnosis	Frequency	Percent
Upper-limb PVD	12	52.2
Upper-limb acute limb ischemia	6	26.0
Upper-limb necrotizing fasciitis	4	17.4
Upper-limb compartment syndrome with PVD	1	4.4
Total	23	100.0

**Figure 3:**
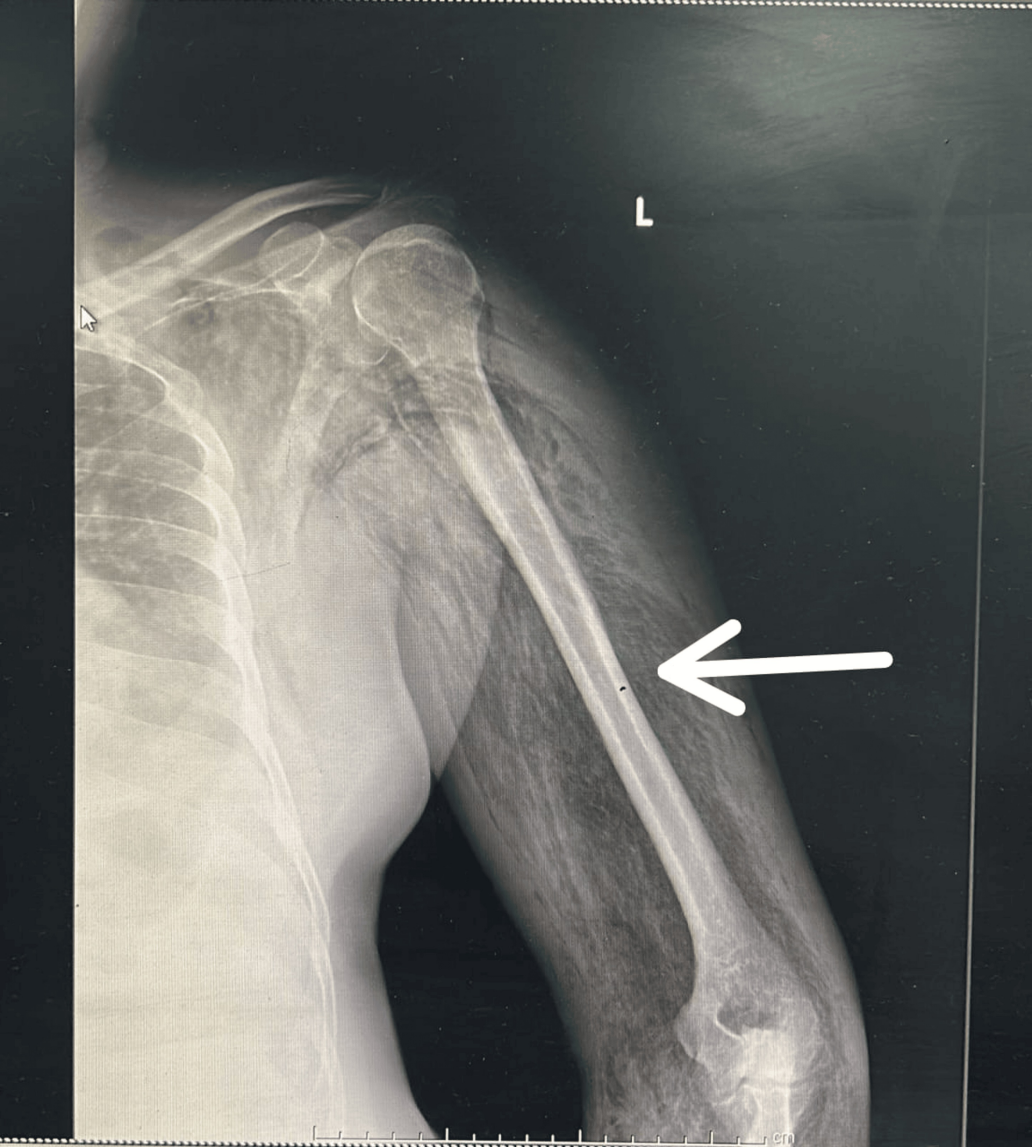
X-ray anteroposterior view of the left shoulder with the arm depicting gas in the muscle planes suggestive of left upper-limb gas gangrene.

**Figure 4:**
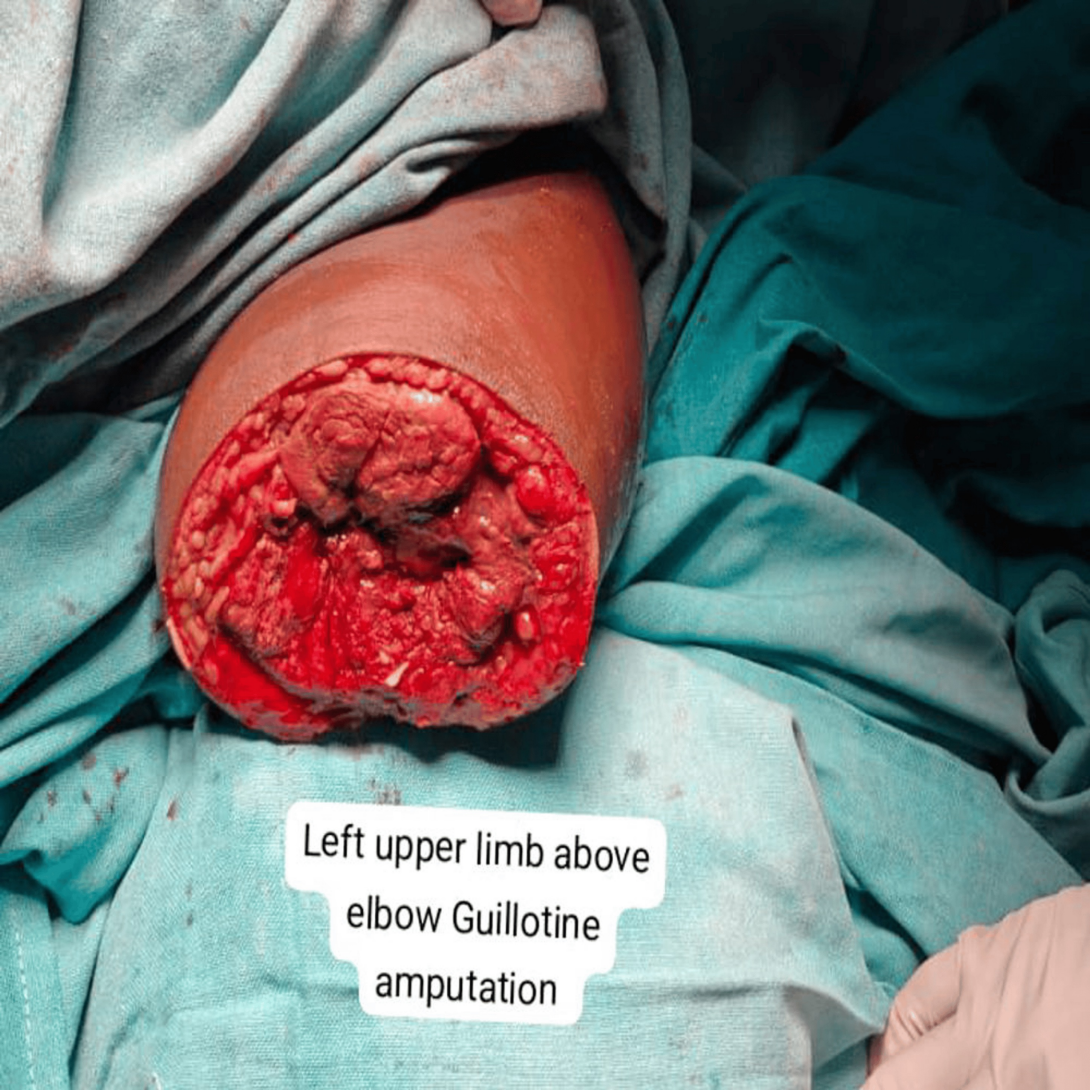
A case of left upper-limb gas gangrene with peripheral vascular disease, after the patient underwent a left-above elbow guillotine amputation.

Figure 3 and Figure 4 depict a case of left upper-limb gas gangrene with peripheral vascular disease where the patient initially underwent left above-elbow guillotine amputation, followed by revision amputation (left shoulder disarticulation in view of muscle necrosis).

Levels of amputation

A vast majority of the individuals underwent above-elbow amputation, as shown in Table 7. Two cases underwent revision amputation, shoulder disarticulation, and above-elbow guillotine amputation in view of muscle necrosis postoperatively. All amputations were performed after vascular surgery consultation.

**Table 7:** Level of amputaion of the study participants.

Level of amputation	N (%)
Above-elbow amputation	21 (91.3%)
Below-elbow guillotine amputation	1 (4.3%)
Above-elbow guillotine amputation	1 (4.3%)

**Figure 5:**
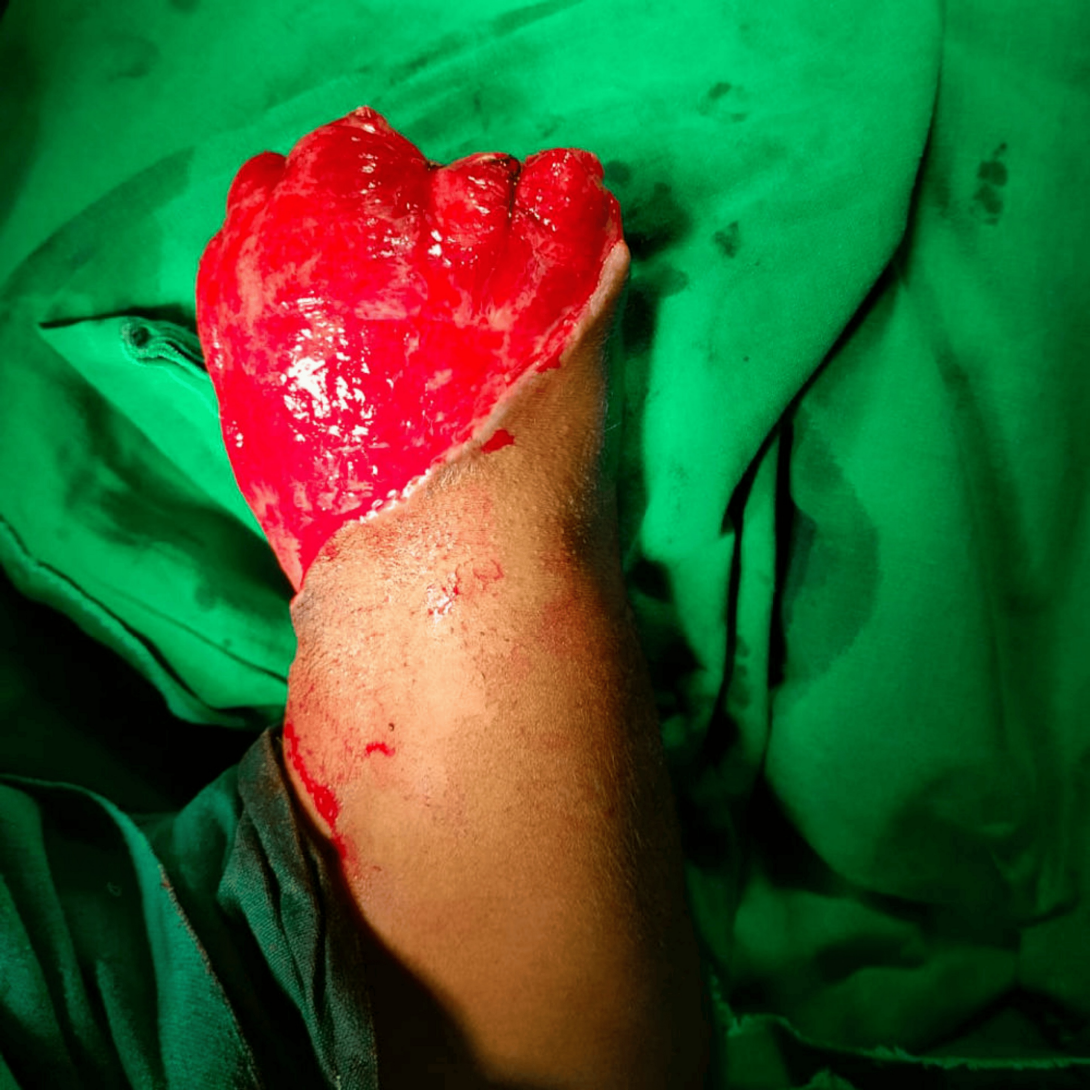
A case of a right above-elbow guillotine amputation in view of right upper-limb necrotizing fasciitis.

Figure 5 depicts a case of right upper-limb necrotizing fasciitis with extensive tissue loss, where initially the patient underwent a right below-elbow guillotine amputation, followed by revision amputation (right above-elbow guillotine amputation in view of stump infection).

Surgical outcomes

Surgical outcomes were analyzed with respect to revision amputation, ICU admissions, surgical site infection, hospital readmission, and mortality. Surgical site infection was classified according to the Centers for Disease Control and Prevention guidelines. Revision amputation was defined as any subsequent surgical procedure to proximalize the amputation level. ICU admissions during the course of the hospital stay were noted. Hospital readmission was tracked for 30 days post-discharge, and mortality was defined as any death occurring during the primary hospital stay (Table 8).

**Table 8:** Surgical outcomes of the study participants.

Surgical outcomes	N (%)
Revision amputation	2 (8.6%)
Intensive care unit admissions	2 (8.6%)
Surgical site infection	2 (8.6%)
Hospital readmission	1 (4.3%)
Death	1 (4.3%)

Duration of hospital stay

The data on the duration of hospital stay for the patients revealed a relatively short and consistent postoperative recovery period, with a mean length of stay of approximately 8.8 days. The stays ranged from a minimum of five days to a maximum of 20 days, but the low standard deviation of 3.5 days indicates that most individual stays were clustered closely around this eight-to-nine-day average, showing little variability.

## Discussion

In this study, we examined the clinical characteristics, comorbidities, and outcomes of patients undergoing major upper-limb amputation for nontraumatic causes. Our findings underscore the dominant role of vascular insufficiency as an etiology, with 78% of cases in our study attributed solely to ischemic pathology. This contrasts sharply with the patterns reported in earlier studies, where infectious causes were more common. For instance, Ting et al. [17], in their 13-year retrospective review of 140 nontraumatic upper-extremity amputations, identified infection as the primary indication, with only 5.7% involving major upper-limb levels. Their cohort demonstrated a median age of 62 years and a predominance of male patients (64%), demographic trends similar to those observed in our population. Diabetes and smoking were major risk factors in both studies, suggesting consistent global patterns in comorbidity profiles. However, the overwhelming burden of vascular pathology in our cohort highlights regional differences in disease epidemiology and access to early vascular care.

The high prevalence of ischemic limb disease in our study likely reflects broader epidemiologic transitions, where diabetes, hypertension, and peripheral arterial disease continue to rise. These comorbidities accelerate atherosclerotic changes and impair microvascular perfusion, predisposing patients to irreversible tissue compromise and delayed wound healing. Several large-scale analyses have reported similar trends, with diabetes and peripheral arterial disease accounting for more than half of nontraumatic amputations worldwide. In our cohort, these conditions were associated not only with limb loss but also with poor postoperative outcomes, reinforcing the need for aggressive medical optimization and early vascular assessment.

Infectious etiologies, although less common in our patient population, remain clinically important. Studies on upper-extremity NSTIs have consistently shown high morbidity and mortality, particularly in patients with diabetes, renal dysfunction, and sepsis at presentation. The psychosocial and functional consequences of major upper-limb amputation are profound, often exceeding those associated with lower-limb loss. Limited access to prostheses, financial constraints, and inadequate rehabilitation infrastructure pose significant barriers to functional recovery in the Indian context. These challenges underscore the importance of preventing major upper-extremity amputations whenever feasible through early multidisciplinary intervention.

Compared with existing literature, our study makes several important contributions. First, it highlights vascular insufficiency as a major cause of nontraumatic major upper-limb amputations within our region. Second, it provides a detailed description of comorbid risk factors and postoperative outcomes, offering insights into modifiable determinants of morbidity. Lastly, our findings emphasize the need for earlier detection and aggressive management of peripheral vascular disease and diabetes to reduce the need for limb-threatening interventions.

This study is limited by its small sample size and retrospective, single-center design. Data on smoking and microbiology patterns were restricted to available clinical records. As a descriptive cohort lacking multivariable modeling, these findings suggest associations rather than causality. Despite these constraints, this study provides valuable descriptive data on a rare clinical entity.

## Conclusions

Vascular insufficiency emerged as the most common cause of nontraumatic major upper-limb amputation in our study. This study provides a descriptive analysis of the clinical presentation and outcomes of patients undergoing major upper-limb amputation for nontraumatic indications at a tertiary care center. Diabetes mellitus and smoking were frequently observed comorbidities within this cohort. While limited by a small sample size and a retrospective design, our findings characterize the common clinical patterns and postoperative complications seen in these patients. These findings reinforce the importance of early recognition and aggressive management of vascular and metabolic risk factors to prevent progression to irreversible tissue loss. Timely vascular assessment, optimization of glycemic control, smoking cessation, and coordinated multidisciplinary care could substantially improve limb-salvage prospects and reduce the morbidity associated with major upper-limb amputation. Strengthening preventive strategies and improving access to early intervention may therefore play a vital role in reducing the burden of nontraumatic upper-extremity amputation in high-risk populations.
